# Whipple’s disease diagnosed during anti-tumor necrosis factor alpha treatment: two case reports and review of the literature

**DOI:** 10.1186/s13256-015-0632-6

**Published:** 2015-07-28

**Authors:** Jose M. Ramos, Francisco Pasquau, Nora Galipienso, Beatriz Valero, Angela Navarro, Agustín Martinez, José Rosas, Ana Gutiérrez, Rosario Sanchez-Martínez

**Affiliations:** 1 Department of Internal Medicine, Hospital General Universitario de Alicante, c/ Pintor Baeza, 12, 03010 Alicante, Spain; 2 Department of Medicine, Miguel Hernández University of Elche, Sant Joan d’Alacant, 03550 Spain; 3 Department of Internal Medicine, Hospital Marina Baixa, Avenida Alcalde Jaume Botella Mayor, 7, Villajoyosa, 03570 Alicante Spain; 4 Department of Rheumatology, Hospital General Universitario de Alicante, c/ Pintor Baeza, 12, 03010 Alicante, Spain; 5 Department of Rheumatology, Hospital Marina Baixa, Avenida Alcalde Jaume Botella Mayor, 7, Villajoyosa, 03570 Alicante Spain; 6 Department of Gastroenterology, Hospital General Universitario de Alicante, c/ Pintor Baeza, 12, 03010 Alicante, Spain; 7 Servicio de Medicina Interna, Hospital General Universitario de Alicante, c/ Pintor Baeza, 12, 03010 Alicante, Spain

**Keywords:** Whipple disease, Infliximab, Etanercept, Spondylarthritis, Rheumatoid arthritis

## Abstract

**Introduction:**

Whipple’s disease is a rare infectious disease caused by *Tropheryma whipplei* with protean clinical manifestations. This infection may mimic chronic inflammatory rheumatisms.

**Case presentation:**

We report two cases of Whipple’s disease diagnosed in the context of an inflammatory disease with anti-tumor necrosis factor alpha failure. The first patient was a 58-year-old white man with psoriatic spondylarthritis, who was treated with adalimumab, etanercept, infliximab, tocilizumab and golimumab. The second was a 73-year-old white man with rheumatoid arthritis, who received treatment with infliximab, then etanercept and rituximab.

**Conclusions:**

Whipple’s disease should be suspected in all patients diagnosed with chronic inflammatory rheumatism, partially controlled or not controlled by treatment with tumor necrosis factor alpha blockers, whose condition worsens after treatment.

## Introduction

Whipple’s disease (WD) is a rare, chronic, systemic infection caused by *Tropheryma whipplei*, a Gram-positive intracellular bacillus related to actinomycetes. WD is a rare infectious disease with protean clinical manifestations. WD may often manifest itself as chronic seronegative oligoarthritis or polyarthritis, which may mimic various joint diseases (rheumatoid arthritis or spondylarthritis). The organism may be detectable by periodic acid–Schiff (PAS) staining of affected organ tissue, especially small bowel, or with 16S ribosomal ribonucleic acid (rRNA) gene identification by polymerase chain reaction (PCR) amplification [1, 2].

Increased susceptibility to infections is a major safety concern with tumor necrosis factor alpha (TNF-α) antagonist treatment [3]. Infection should be ruled out in atypical cases by searching for foci [4]. Several authors have shown that treatment with TNF-α blockers seems to increase the risk of exacerbating WD [5] or worsening preexisting WD, triggering visceral disorders [4, 6].

We describe two cases of patients who were given TNF-α antagonists to treat long-standing joint disease, who then experienced the involvement of several organs leading to the diagnosis of WD. We also review the case reports of WD diagnosed during anti-TNF-α treatment.

## Case presentation

From 2000 to 2012, eight cases of WD were diagnosed at two Spanish hospitals (Hospital General Universitario de Alicante, Alicante and Hospital Marina Baixa, Villajoyosa, Alicante), and two cases were associated with use of TNF-α antagonists.

### Case report 1

**Fig. 1:**
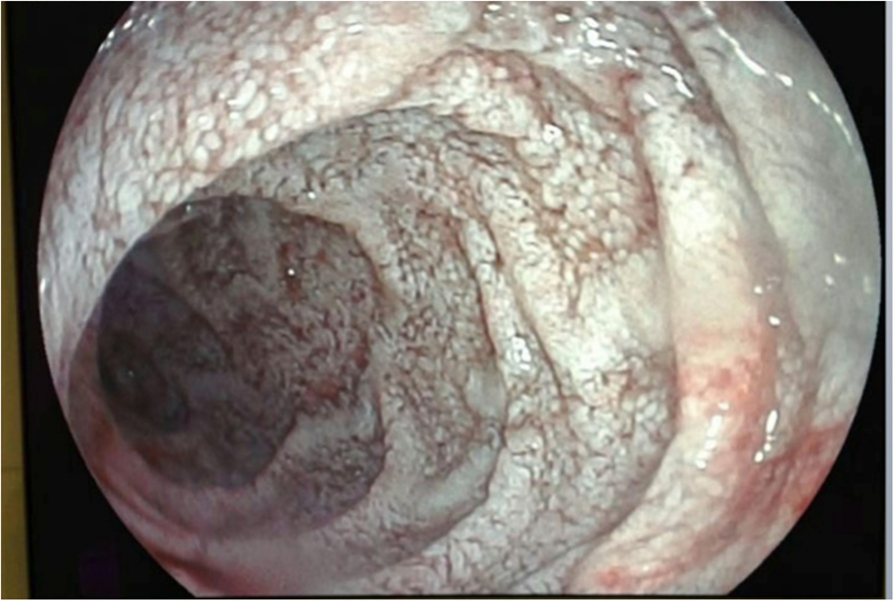
Endoscopy. White lesions compatible with diffuse intestinal lymphangiectasia

A 58-year-old white man with inflammatory back pain and large and small joint arthritis had been diagnosed with psoriatic spondylarthritis 9 years ago. Our patient had been treated with adalimumab for 4 months, after that with etarnecept for 8 months, then infliximab for 2 months, tocilizumab for 21 months and golimumab for 1 month, to treat the pain in his back and neck with the consequent difficulty in bending, and arthritis of his knee and interphalangeal joint arthritis. Our patient was admitted to the hospital with abdominal septic shock. A computed tomography (CT) scan showed multiple retroperitoneal lymph nodes. The colonoscopy result was normal and the biopsy result was normal. Three months later, he was admitted again with a fever and heart failure, which was interpreted as a side effect of the golimumab treatment. One year after that, he was admitted to the hospital with abdominal pain, diarrhea and weight loss progressing to a severe wasting syndrome. At this time, he was being treated with 5mg prednisone plus hydroxicloroquine and methotrexate (MTX). Abnormal laboratory test results included a white blood cell (WBC) count of 14,630/mm^3^, a hemoglobin level of 9.6g/dL and an erythrocyte sedimentation rate (ESR) of 58mm/h. A CT scan showed multiple lymph nodes. Endoscopy showed diffuse intestinal lymphangiectasia (Fig. 1). A duodenal biopsy showed distortion of the villous architecture with abundant macrophages, and bacilliform intracytoplasmic structures that stained positive with PAS with diastase digestion compatible with WD. A PCR assay for detecting *T. whipplei* was not done. Intravenous ceftriaxone (2g daily for 2 weeks) was commenced followed by trimethoprim and sulphamethoxazole with improved symptoms after 3 weeks; treatment was continued for 18 months. One year later, a new gastroscopy with duodenal biopsy was done. It did not show intestinal lymphangiectasia. A PCR assay result for *T. whipplei* was negative. There were no relapses after 19 months.

### Case report 2

A 73-year-old white man had been diagnosed with rheumatoid arthritis with migratory arthralgias of the large joints and chronic obstructive pulmonary disease 14 years ago. Our patient had been treated with gold salts, chloroquine and MTX. Fourteen years after diagnosis of his diseases, infliximab was added to the MTX treatment without improvement, so infliximab was suspended 8 months later because there was no improvement of his migratory nondeforming polyarthritis; treatment with MTX was continued. After 5 months infliximab was stopped, and etanercept was added to MTX for 6 months. During treatment with etanercept, he suffered an acute middle cerebral artery ischemic stroke of atherothrombotic origin, and etanercept was stopped. Six months later, rituximab was added for 3 months, without improvement. After that, MTX was stopped and leflunomide (20mg/day) was initiated and from that point, our patient presented with abdominal pain, chronic diarrhea and edema in his lower extremities, a consequence of chronic malabsorption. After 1 year on this treatment, he was admitted to hospital with rectal bleeding, however, the colonoscopy and gastroscopy results were normal and the colon biopsy showed unspecific changes. At that time, our patient was being treated with leflunomide, which was then stopped. Three months after that admission, our patient was admitted with weight loss, abdominal pain and diarrhea. On physical examination, he had hyperpigmentation of the skin but no other abnormalities. Abnormal laboratory test results included a WBC count of 13,800/mm^3^, a hemoglobin level of 9.2g/dL, mean corpuscular volume (MCV) of 72fl, an albumin level of 1.8g/dL, and an ESR of 13mm/h. A thoracic and abdominal CT scan showed pericardial effusion with calcifications, bronchiectasis in his lower right lung, intestinal bowel with distention and no abdominal lymph nodes. A duodenal biopsy showed altered architecture and intracellular bacilli on PAS stain. *T. whipplei* was detected from duodenal tissue by PCR assay. A cerebral magnetic resonance imaging scan showed multiple hyperintensive lesions in both cerebral hemispheres, cortical retraction, increased subarachnoid space and ventricular dilatation. The PCR assay result for *T. whipplei* in his cerebrospinal fluid was negative. Intravenous ceftriaxone (2g daily) was commenced for 2 weeks followed by trimethoprim and sulphamethoxazole with improvement of his symptoms (the diarrhea, malabsorption and pericardial effusion). One year later, a new gastroscopy with duodenal biopsy was done. It showed altered architecture and intracellular bacilli on PAS stain, but the PCR assay result for *T. whipplei* was negative. Because of mild renal failure, trimethoprim and sulphamethoxazole was changed for doxycycline plus hydroxychloroquine, and normal renal function was recovered.

## Discussion

We reviewed database cases recorded in PubMed using the following retrieval scheme: [“Whipple disease” and (“infliximab” or “adalimumab” or “etanercept” or “golimumab” or “tocilizumab”)]. We gathered the following data from the medical cases reported: age, sex, joint diseases, years with joint disease, TNF-α antagonist therapy, days with TNF-α antagonist therapy before WD was diagnosed, symptoms related to WD, organs affected by WD, investigations for diagnosing WD, treatment and outcome of WD.

We retrieved 14 cases from the PubMed database from January 2004 to December 2014. All the case reports recorded and the two case reports in this manuscript are from European scientists [4–10], except one case from the United States of America [11]. Four case reports were published in a language other than English [7, 8, 10]. Table 1 shows age, sex, joint diseases, years with joint disease, TNF-α antagonist therapy, days with TNF-α antagonist therapy before WD was diagnosed, symptoms related to WD, organs affected by WD, investigations for diagnosing WD, treatment and outcome of WD for 16 WD cases. Out of 16 cases, there were 14 men and two women patients with an age range of 33 to 73 years old. Six patients were diagnosed as ankylosis spondylitis (AS), six as seronegative spondyloarthropathy (SA) (seronegative chronic polyarthritis), two as rheumatoid arthritis (RA), one as Still’s disease (SD), and one as psoriatic arthritis. All patients had osteoarticular (with pain and swelling) involvement and 15 had gastrointestinal involvement (diarrhea, weight loss, abdominal pain, malabsorption, and so on) [4–12]. Moreover, some patients had extra-articular and gastrointestinal involvement, such as vertebra [5], meningitis [6], pericarditis ([6], present report 2 (PR2)), abdominal or thoracic lymph nodes ([4, 6], PR1), gingiva such as scurvy [9] and heart valve involvement [10]. All were diagnosed by histological biopsy, in 14 of 15 cases a PCR assay for DNA detection of *T. whipplei* was done, and all were positive. The DNA of *T. whipplei* by PCR assay was detected in duodenal or other gastrointestinal-colonic areas; saliva, blood, feces, bone or cerebrospinal fluid [4–11]. All patients recovered from gastrointestinal involvement when the TNF-α antagonist was stopped and antibiotic treatment was started.

**Table 1:** Features of the patients’ diagnosis of Whipple’s disease after tumor necrosis factor alpha antagonist initiation

Authors / country / language of publication	Age / sex	Underlying disease previous WD diagnosis / time with that diagnosis	TNF-α drugs and immunosuppressive treatment	Start of acute symptoms after TNF-α blockade	Symptoms after TNF-α blockade	Organ involvement	Histological diagnosis^a^	Result of PCR for *Tropheryma whipplei*	Treatment for WD	Outcome
Kneitz *et al*. (2005) [4] / Germany / English	34y / M	SD	Methotrexate + infliximab (2w)	2w	Weight loss, erythema nodosum, diarrhea, lymph node enlargement, and a sigmoidovesical fistula	Skin, gastroduodenitis and sigmoidovesical	Positive in fistula and lymph nodes	Positive in lymph node, small bowel and sigmoidovesical fistula	SXT (12m)	Resolved within 1 year
Kremer *et al*. (2008) [7] / Germany / German	47y / M	SA / 4y	Leflunomide + adalimumab		Fever, weight loss, and severe arthralgia		Positive in duodenal tissue	Positive in duodenal tissue	SXT	
Spoerl *et al*. (2009) [5] / Switzerland / English	64y / M	SA / 5 + 3y	Etanercept	4m	Lethargy, night sweats, weight loss	Gastrointestinal and vertebral	Positive in duodenal tissue and vertebral tissue	Positive in gastric and vertebral tissue	Ceftriaxone (2w), then, SXT (2y), (12m) then doxycycline + HC (18m)	No relapses after 34-month follow-up
Ahmadi-Simab *et al*. (2009) [8] / Germany / German	33y / F	AS / 8y	Etanercept	12m	Diarrhea, abdominal pain and weight loss		Negative in duodenal tissue	Positive in duodenal biopsy	Ceftriaxone (2w) after SXT	
Hoppé *et al*. (2010) [6] / France / English	67y / F	SA / 7y	Infliximab (4m)	4m	Chest pain, dyspnea, polyarthritis		Positive in duodenal tissue	Positive in duodenal tissue. Negative in blood and saliva	Ceftriaxone (2w), then doxycycline (24m)	Resolved within 15d. No relapses after 71-month follow-up
Hoppé *et al*. (2010) [6] / France / English	40y / M	AS / 1y	Infliximab (18m) then etanercept (7m) then adalimumab (1m)	26m	Diarrhea, weight loss, fever, arthralgia	Widespread ileocolitis, gastroduodenitis, meningitis	Positive in duodenal tissue	Positive in saliva, feces and cerebrospinal fluid. Negative in blood	Doxycycline, HC, and SXT (15d), then SXT was replaced by sulfasalazine 4g/day	Resolved within 2 months. No relapses after 17-month follow-up
Hoppé *et al*. (2010) [6] / France / English	60y / M	AS / 8y	Etanercept (9m)	9m	Fever, night sweats, polyarthritis, chest pain	Pericarditis, mediastinal and abdominal lymphadenopathy, duodenitis	Negative in duodenal tissue	Positive in the duodenal sample, saliva, feces, and lymph nodes	Doxycycline + HC (15m)	Resolved within 7d. No relapses after 30-month follow-up
Hoppé *et al*. (2010) [6] / France / English	47y / M	RA / 17y	Infliximab (36m), then etanercept (42m), then adalimumab (6m), then rituximab, abatacept then infliximab (1m)	85m	Fever, night sweats, weight loss, polyarthritis, radio carpal arthropathy. transient diplopia, myalgia, subacute depression	Pericarditis, abdominal lymphadenopathy, duodenitis		Positive PCR assay in the duodenal sample, blood, saliva, and feces. Negative PCR assay in cerebrospinal fluid	Doxycycline + HC (UT)	Resolved within 2m No relapses after 15-month follow-up
Hoppé *et al*. (2010) [6] / France / English	38y / M	AS / 9y	Etanercept (2m)	9m	Diarrhea, abdominal pain. weight loss, blurred vision	Hemorrhagic gastroduodenitis, hemorrhagic colitis, mediastinal and cervical lymphadenopathy, splenomegaly, meningitis	Positive in duodenum and colon	Positive PCR assay in blood, saliva, cerebrospinal fluid, and feces	Doxycycline 200mg/day. HC 600mg/day. (UT)	Resolved within 3w. No relapses after 13-month follow-up
Hmamouchi *et al*. (2010) [9] / France / English	35y / M	AS / 4y	Etanercept	4m	Gingival nodule, purpura, abdominal pain diarrhea, fatigue, weight loss	Gingiva and colitis	Positive in colon tissue	Positive in blood, stool, and cerebrospinal fluid	SXT (NR)	Resolved within 3 months
Daïen *et al*. (2010) [10] / France / English	70y / M	SA / 27y	Etanercept (18m)	NR	Fever and dyspnea	Endocarditis of aortic valve	Negative in duodenal tissue and, aortic valve	Positive in aortic valve	Doxycycline HC + SXT (18m)	Resolved. No relapses after 21-month follow-up
Gaddy *et al*., (2012) [12] / USA / English	46y / M	AS / 10y	Prednisone + MTX + infliximab (24m), then adalimumab	24m	Fever, migratory arthritis, weight loss, diarrhea	Gastroenteritis	Positive in duodenal tissue	Positive in blood and duodenal tissue	Ceftriaxone (x2w) after SXT	NR
Sparsa *et al*. (2013) [11] / France / French	53y / M	SA	Etanercept (3m), then adalimumab (6m)	9m	Polyarthralgia peripheric, arthritis metarsophalangeal		Positive in duodenal tissue biopsy	Positive in saliva and duodenal tissue	Doxycycline + HC (18m)	Resolved within 3 weeks. No relapses after 18-month follow-up
Sparsa *et al*. (2013) [11] / France / French	42y / M	SA / 2m	Adalimumab (9m) and then etanercept (4m)	13m	Polyarthralgia peripheric		Positive in duodenal and gastric tissue	Positive in saliva and duodenal tissue	Doxycycline + HC and then doxycycline alone	Resolved within 3 weeks. No relapses after 17- month follow-up
Present report	58y / M	Psoriatic spondyloarthropathy/ 14y	Adalimumab (4m), then etanercept (8m), infliximab (2m), tocilizumab (21m), golimumab (1m)	36m	Abdominal pain, diarrhea and weight loss	Duodenitis and abdominal lymph nodes	Positive in duodenal tissue	Not done	Ceftriaxone (14d), then SXT during (UT)	Resolved within 3 weeks. No relapses after 9-month follow-up.
Present report	78y / M	RA / 19y	Infliximab (6m), etanercept (6m), rituximab (3m), leflunomide (24m)	24m	Abdominal pain, diarrhea and weight loss	Duodenitis, and pericardial effusion	Positive in duodenal tissue	Positive in duodenal biopsy. Negative in cerebrospinal fluid	Ceftriaxone (14d), then SXT for (12m) then doxycycline + HC	Resolved within 3 weeks. No relapses after 18-month follow-up

(UT) Patient still under treatment

*TNF-α* tumor necrosis factor alpha, *WD* Whipple’s disease, *PCR* polymerase chain reaction, *y* year, *SD* Still’s disease, *w* week, *SXT* cotrimoxazole, *m* month, *SA* spondyloarthropathy (seronegative chronic polyarthritis), *HC* hydroxychlorochine, *AS* ankylosis spondylitis, *RA* rheumatoid arthritis, *NR* not reported, *MTX* methotrexate

^a^Histological diagnosis = intracellular bacilli on periodic acid–Schiff stain

## Conclusions

WD is a systemic infection, which involves many chronic manifestations, especially digestive disorders. Some of the manifestations involved are articular symptoms, which can appear as inflammatory rheumatism, such as rheumatoid arthritis or spondylarthritis. Beside these symptoms, the microorganism responsible for *T. whipplei* disease has also been found in DNA recovered from articular fluid and bones of these patients [13, 14].

Our two cases illustrate that alternative etiologies in patients with articular symptoms should be considered, most importantly following clinical deterioration by immunomodulatory agents like TNF-α inhibitors. The reflections to be made with respect to the analysis of these two cases are to speculate that these patients either had WD with mainly articular manifestations, which had been considered as a rheumatic disease; or in fact, they had a rheumatic disease, which, through the treatment with TNF drugs, may have reactivated *T. whipplei* and the appearance, therefore, of its florid symptoms that we managed to diagnose.

Therapies with immunomodulators, TNF-α inhibitors, and corticosteroids may transform an infection with *T. whipplei*, normally at a subacute stage, into a septic, life-threatening disease. Thus, it seems that TNF-α blockade allowed for rapid dissemination of *T. whipplei* by inhibiting some important immune defense mechanisms. In the later phase of an infection, the TNF-α blocker contributes to limiting the extent of cell and/or tissue damage by inducing apoptosis and maintaining granuloma formation [15, 16], which are important mechanisms in *T. whipplei* infections. TNF-α blockade might result in the loss of some of the immunological control mechanisms and, therefore, facilitate rapid bacterial dissemination and severe exacerbation of the disease. This is the situation in the first case.

In the second case, the TNF-α blockade was used before the gastrointestinal symptoms appeared. However, with anti-TNF-α treatment, our patient had an acute stroke. After stopping the treatment, our patient recovered from his central nervous system symptoms. When the gastrointestinal involvement of WD was discovered, an examination of DNA for *T. whipplei* was performed but it was negative. In this case, it was not clear that our patient had neurological involvement of WD, and real improvement of WD after stopping the TNF-α blockade treatment was not clear. There are several cases reported of patients with rheumatic diseases and, during follow-up, they were diagnosed with WD [13, 14, 17, 18]. Thus, the case report of a patient with an initial diagnosis of rheumatoid arthritis who developed pericarditis caused by WD (diagnosed by pericardial biopsy) and the patient who had not been treated previously with TNF-α blocker [17].

In summary, anti-TNF-α treatment seems to increase the risk of exacerbation of WD and WD may mimic a rheumatic disease.

## Consent

Written informed consent was obtained from the patients for publication of this case report and any accompanying images. A copy of the written consent is available for review by the Editor-in-Chief of this journal.

## Abbreviations

AS: Ankylosis spondylitis
CT: Computed tomography
ESR: Erythrocyte sedimentation rate
HC: Hydroxychlorochine
m: month
MCV: Mean corpuscular volume
MTX: Methotrexate
NR: Not reported
PAS: Periodic acid–Schiff
PR: Present report
PCR: Polymerase chain reaction
RA: Rheumatoid arthritis
SA: Spondyloarthropathy (seronegative chronic polyarthritis)
SD: Still’s diseases
SXT: Cotrimoxazole
rRNA: ribosomal ribonucleic acid
TNF-α: Tumor necrosis factor alpha
w: week
WBC: White blood cell
WD: Whipple’s disease
y: year
